# Tackling Childhood Stunting in the Eastern Mediterranean Region in the Context of COVID-19

**DOI:** 10.3390/children7110239

**Published:** 2020-11-19

**Authors:** Ayoub Al Jawaldeh, Radhouene Doggui, Elaine Borghi, Hassan Aguenaou, Laila El Ammari, Azza Abul-Fadl, Karen McColl

**Affiliations:** 1 WHO Regional Office for Eastern Mediterranean Region, Cairo 11371, Egypt; 2 Department of Family Medicine, Université de Sherbrooke, Sherbrooke, QC J1K 2R1, Canada; radhouene.doggui@usherbrooke.ca; 3 Centre de Formation méDicale du Nouveau-Brunswick, Moncton, NB E1A 7R1, Canada; 4 WHO Department of Nutrition and Food Safety, Geneva, 1211 Geneva, Switzerland; borghie@who.int; 5 Joint Unit of Research in Nutrition and Food, RDC-Nutrition AFRA/IEA, Ibn Tofail University-CNESTEN, Kénitra 14000, Morocco; AGUENAOU.Hassan@uit.ac.ma; 6 Programme National de Nutrition, Ministère de la Santé, Rabat 10090, Morocco; elammarilaila0@gmail.com; 7 Benha Faculty of Medicine, Benha University 26B, Cairo 11211, Egypt; azza_abulfadl@yahoo.com; 8 Karen McColl Consulting, West Sussex PO21 4NB, UK

**Keywords:** nutrition, childhood malnutrition, stunting, Eastern Mediterranean, Near East, North Africa, COVID-19, coronavirus

## Abstract

Over 20 million children under 5 years old in the WHO Eastern Mediterranean Region have stunted growth, as a result of chronic malnutrition, with damaging long-term consequences for individuals and societies. This review extracted and analyzed data from the UNICEF, WHO and the World Bank malnutrition estimates to present an overall picture of childhood stunting in the region. The number of children under 5 in the region who are affected by stunting has dropped from 24.5 million (40%) in 1990 to 20.6 million (24.2%) in 2019. The reduction rate since the 2012 baseline is only about two fifths of that required and much more rapid progress will be needed to reach the internationally agreed targets by 2025 and 2030. Prevalence is highest in low-income countries and those with a lower Human Development Index. The COVID-19 pandemic threatens to undermine efforts to reduce stunting, through its impact on access and affordability of safe and nutritious foods and access to important health services. Priority areas for action to tackle stunting as part of a comprehensive, multisectoral nutrition strategy are proposed. In light of the threat that COVID-19 will exacerbate the already heavy burden of malnutrition in the Eastern Mediterranean Region, implementation of such strategies is more important than ever.

## 1. Introduction

Chronic childhood malnutrition (stunting) remains a major challenge in the World Health Organization (WHO) Eastern Mediterranean Region (Afghanistan, Bahrain, Djibouti, Egypt, Islamic Republic of Iran, Iraq, Jordan, Kuwait, Lebanon, Libya, Morocco, Oman, occupied Palestinian territory, Pakistan, Qatar, Kingdom of Saudi Arabia, Somalia, Sudan, Syrian Arab Republic, Tunisia, United Arab Emirates, Yemen). Millions of children under 5 years old in the region have had their growth stunted by chronic malnutrition, with serious lifelong consequences for their health and development [1]. Robust action continues to be needed to reduce this major barrier to human development, particularly as the COVID-19 pandemic presents a new threat to food security and nutrition in the region [2].

Stunting is a chronic form of malnutrition, whereby a child is stunted if they are too short for their age, according to the WHO child growth standards [3]. Stunting is the largely irreversible outcome of poor nutrition and infection during the first 1000 days from conception to a child’s second birthday [4].

Childhood stunting is associated with increased child mortality and morbidity and has long-term effects on individuals, through diminished cognitive and physical development, poor health and increased risk of noncommunicable diseases (NCDs) later in life [5,6,7,8]. Stunting also has serious consequences for societies, through loss of physical growth potential, cognitive impairments, reduced productive capacity, loss of economic productivity and the healthcare costs associated with the poorer health of stunted individuals [4,8,9]. Stunting is an enormous drain on economic productivity and growth—stunted individuals have reduced productive capacity and it is estimated that a 1% loss in adult height due to stunting is associated with a 1.4% loss in economic productivity and that stunting can reduce a country’s gross domestic product (GDP) by up to 3% [10,11]. Scaling-up action to address stunting has been described as one of the best investments that countries can make to help generate and sustain broad-based wealth [8].

The countries of the Eastern Mediterranean Region have signed up to global and regional targets to reduce the numbers of children under 5 years of age that are stunted. These include the 2012 World Health Assembly global target for a 40% reduction by 2025 [12], from a 2012 baseline of 21.4 million, and a proposed global and a regional target for a 50% reduction by 2030 [13,14].

**Figure 1:**
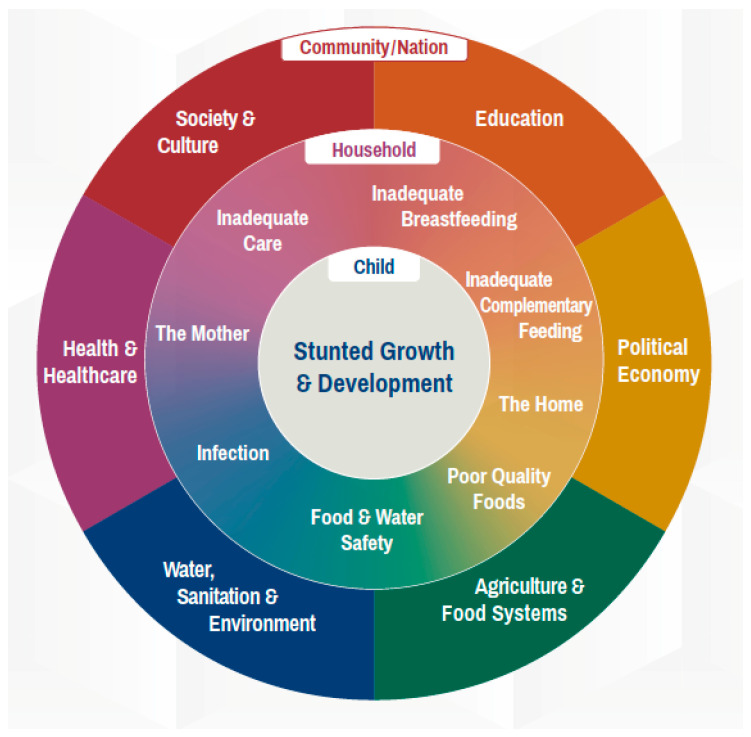
Stunted growth and development: context and causes [15].

Many different factors—at community, household and family levels—can contribute to childhood stunting, as summarized in the conceptual framework shown in Figure 1 [15].

At the household level, there are many causes which can contribute to stunting, including inadequate sanitation and water supply, low wealth and socioeconomic status, food insecurity, low status of women, poor caregiver education, inappropriate intra-household food allocation, poor-quality foods, contaminated food and water and infection [7]. Other contributing factors include poor maternal nutrition and inadequate care, breastfeeding or complementary feeding [7,16]. There are many aspects of the wider context that can contribute, including food prices and trade policy, marketing regulations, political stability, poverty, access to healthcare, agriculture and food systems, education, society and culture, as well as aspects of the environment [4].

It is clear that many different sectors—involving people from a variety of disciplines—need to be involved in designing effective strategies at the national, regional, community and household levels for the prevention of stunting. International policy guidance reflects the multifactorial nature of malnutrition and the importance of multisectoral solutions. A comprehensive implementation plan on maternal, infant and young child nutrition was adopted by the World Health Assembly in 2012, setting out the global nutrition targets for 2025 [12]. The Second International Conference on Nutrition in 2014 adopted a Framework for Action which provides a set of 60 recommended policies and actions [17]. To further drive progress in this area, the United Nations General Assembly declared a UN Decade of Action on Nutrition for the period 2016–2015 [18]. Specifically in relation to stunting, WHO has published two policy briefs with guidance on how to drive progress towards the global target [4] and how to tackle stunting with a focus on equity [19], as well as action points for implementing a stunting reduction agenda [20]. A multisectoral approach to tackling malnutrition also underpins the WHO Strategy on nutrition for the Eastern Mediterranean Region 2020–2030, which was adopted in October 2019 to support countries to strengthen efforts to ensure universal access to healthy and sustainable diets and implement effective nutrition actions [13].

This review presents an up-to-date picture of child stunting in the countries of the Eastern Mediterranean Region. It examines the regional trend in stunting over recent decades, summarizes the situation in the countries in the region and examines the association between stunting prevalence and levels of income and human development. It also summarizes data on the implementation of a multisectoral approach to tackling malnutrition in the region. A number of key areas for policy action are highlighted to drive progress in this area. 

## 2. Materials and Methods

Global and regional estimates of stunting prevalence have been published by the United Nations Children’s Fund (UNICEF), WHO and the World Bank in joint malnutrition estimates since 2012, with the most recent edition published in March 2020 [1]. These data are based on available national survey data, mainly from population-based household surveys with anthropometry and nutrition surveillance systems. Data are collected in line with the operational guidance for the Global Nutrition Monitoring Framework [21] and the methodology is described elsewhere [1]. National survey results are evaluated for inclusion in the joint analysis dataset. Prevalence estimates are based on the 2006 WHO child growth standards, and stunting figures refer to the proportion of children with height-for-age below two standard deviations under the median [3]. The estimates are adjusted where necessary to be nationally representative and to cover the age range 0–5 years. This and other adjustments based on the recommended standard analysis are made to ensure comparability of results, and these estimates are not necessarily comparable with values reported in national surveys. The detailed methodology used for deriving regional and global trends has been published elsewhere [22].

Within the Global Nutrition Monitoring Framework, monitoring rules have also been established by the WHO/UNICEF technical expert advisory group on nutrition monitoring (TEAM) to assess country progress towards the global targets [23]. This progress is reported in the Global Nutrition Report series [24].

For this paper, the data on Eastern Mediterranean Region countries were extracted from the database of the 2020 joint malnutrition estimates prepared by UNICEF, WHO and the World Bank Group. Data over time were extracted and a regional trend was plotted. The data extraction and the tabular presentation were conducted by one co-author and then verified by a different co-author. The figures were prepared by one co-author and were also verified by a different co-author.

The World Bank classification was used in order to identify the income level of each country [25]. Currently, the low-income group includes Afghanistan, Somalia, Sudan, Syrian Arab Republic and Yemen. The lower middle-income group includes Djibouti, Egypt, Morocco, Pakistan, Tunisia and occupied Palestinian territory (West Bank and Gaza). The upper middle-income group includes Iran, Iraq, Jordan, Lebanon and Libya. The high-income level includes Bahrain, Kuwait, Oman, Qatar, Saudi Arabia and United Arab Emirates. For the analysis of stunting prevalence by country income level, the income level for the year of data collection in each country was used.

The Human Development Index (HDI) is a proxy of country development. The HDI is constructed by compiling indicators of three dimensions: health (life expectancy at birth), education (years of schooling) and standard of living (per capita gross national income) [26]. The HDI value pertaining to the year of the stunted data collection was retained for the analysis. HDI ≥ 0.80 corresponds to very high development; HDI ≥ 0.70 corresponds to high development; HDI ≥ 0.6 corresponds to medium development; and HDI < 0.6 corresponds to low development.

In addition to the data on the stunting situation in the region, some data on country implementation of multisectoral nutrition strategies, policies and interventions were extracted from the dataset for the second Global Nutrition Policy Review 2016–2017 (GNPR) [27]. The GNPR data are based on a comprehensive survey of nutrition-related policies undertaken between July 2016 and December 2017, to which 176 WHO Member States and one area responded. For this paper, data on the Eastern Mediterranean Region were extracted from the Global Nutrition Policy Review 2016–2017 dataset by one co-author and cross-checked by two other co-authors. On the basis of existing international policy guidance [4,13,17,19,28], priority areas for policy action to tackle stunting as part of a comprehensive, multisectoral nutrition strategy are proposed.

Furthermore, nutrition counterparts and WHO representatives of the countries in the region provided information on the implementation of recommended policy action and two case studies are thus described in the Discussion section of the paper.

## 3. Results

The data analysis presents a picture of the current situation relating to stunting in the Eastern Mediterranean. The extent of country implementation of multisectoral strategies to tackle malnutrition is also summarized.

### 3.1. Childhood Stunting in the Eastern Mediterranean Region

**Figure 2:**
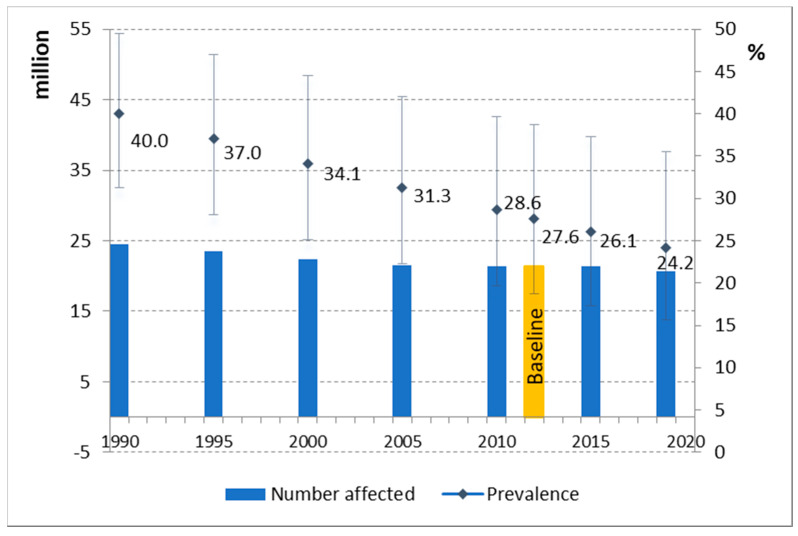
Stunting in the Eastern Mediterranean Region, 1990 to 2019—percentage prevalence and number of children affected (million).

In 2019, the growth of 20.6 million children, nearly one in four (24.2%) of all children in the region, was stunted [1]. The prevalence of stunting has declined significantly since 1990 from 40% to 24.2% in 2019 (Figure 2). The number of children affected has fallen from 24.5 million in 1990 to 20.6 in 2019. Nonetheless, the rate of stunting remains too high and the region is not currently on track to meet the global target of a 40% reduction in the number of stunted children by 2025 [29] or the regional target of a 50% reduction by 2030 [13].

This figure hides the considerable variation across the region. Table 1 shows the baseline and latest available prevalence data for the countries in the region, according to the UNICEF/WHO/World Bank joint malnutrition estimates. As mentioned in the description of the methodology, due to adjustments and/or the standard methodology for the analysis of the anthropometric data applied to ensure comparability of results, these estimates may differ from the values reported in national surveys. The average annual rate of reduction—both the current rate and the rate prior to the baseline level for the 2025 targets—and the rate required to reach the 2025 target are also shown.

According to the categorization of countries according to the level of public health significance as defined by WHO and endorsed by the TEAM [31], considering only countries which have recent data (2015 or later), two countries in the region that have *very high* prevalence of stunting (greater or equal to 30%—Afghanistan and Pakistan) and three that have *medium* levels of stunting (between 10% and 20%—Morocco, Oman and Iraq) were identified.

**Figure 3:**
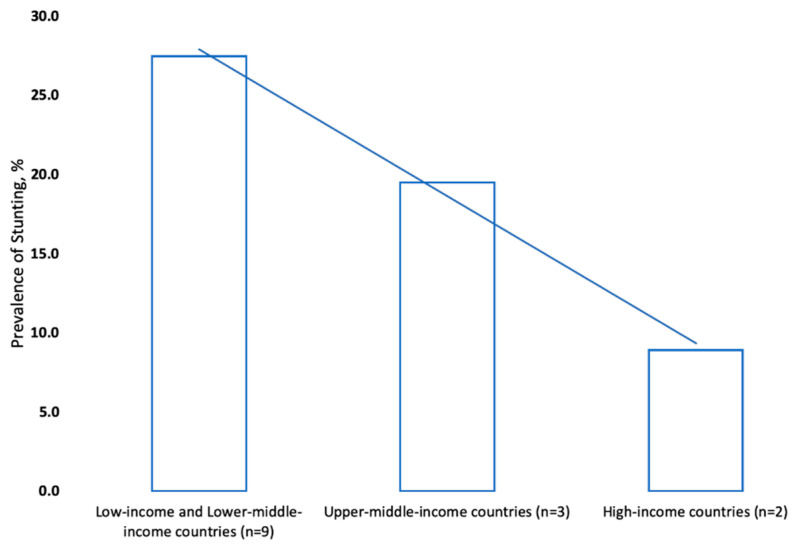
Average stunting prevalence (latest estimates) in countries of different income levels in the WHO Eastern Mediterranean Region ^1,2,3^. ^1^ Latest estimates only include data from 2012 or later. ^2^ Country level of income, by World Bank classification, relates to year of stunting data collection. Low- and lower middle-income countries: Afghanistan (low-income); Djibouti, Egypt, Morocco, Pakistan, Sudan, Tunisia, occupied Palestinian Territory and Yemen; upper middle-income countries: Iraq, Jordan and Libya; high-income countries: Kuwait and Oman. ^3^ Source: analysis based on country data [30].

Mean stunting prevalence was highest in low-income countries (38.2%) compared to lower middle-income countries (26.1%), upper middle-income countries (19.5%) and high-income countries (8.9%), and the inverse relationship is shown in Figure 3.

**Figure 4:**
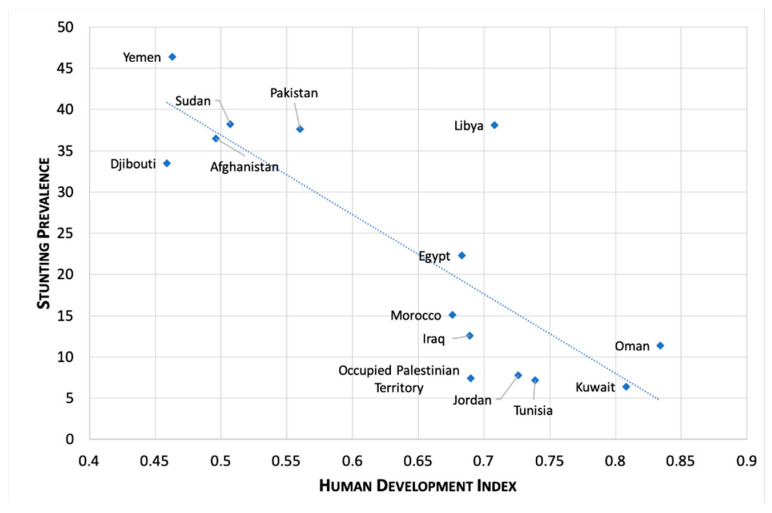
Prevalence of stunting (latest estimates) and Human Development Index (HDI) in the countries of the WHO Eastern Mediterranean Region ^1,2,3^. ^1^ Latest estimates only include data from 2012 or later. ^2^ HDI for each country relates to year of data collection. ^3^ Source: analysis based on country data [30].

There is an inverse association between the level of human development level in a country (HDI) and the prevalence of stunting (Figure 4). 

In terms of progress towards the global nutrition target of a 40% reduction in the numbers of children affected by stunting between 2012 and 2025, only 11 countries in the region had sufficient quality data to assess progress (Table 1). For countries’ progress assessment, the methodology recommended by the TEAM [23] can be applied—sufficient data for calculating AARR and at least one point beyond 2012. Only two countries, Iraq and the occupied Palestinian Territory, are on track to reach the 2025 target of a 40% reduction, with a current rate of reduction of 7.7 and 9.2 percent per year on average compared to the required of 5.3 and 4.8, respectively. Afghanistan, Egypt, Pakistan and Tunisia show some progress but insufficient to reach the target. Compared to the pre-baseline period (1999–2012), Egypt and Pakistan had major improvement, reversing the previously increasing trends. Rates have stagnated in Morocco and Yemen, the latter at a very high level of significance (46.4% in 2013). Kuwait, Oman and Sudan are worsening, that is, increasing their stunting rates, in the most recent years. 

Behind the national prevalence figures, some population groups will be affected more than others, due to different underlying socioeconomic, cultural and ecological factors as well as issues relating to infrastructure and access to food, health, education and social services. It is important that strategies to address stunting consider the extra efforts that are needed to meet the needs of these vulnerable groups.

### 3.2. Multisectoral Policy Responses to Prevent Stunting

In relation to the implementation of a multisectoral approach to tackling stunting and other forms of malnutrition, by 2016/17 the vast majority (89%) of the countries in the region had a comprehensive or topic-specific nutrition policy, and, of those, maternal, infant and young child nutrition was included in 85% of cases [27]. More recently, a number of Member States have published new policy documents relating to nutrition [32,33,34,35,36,37].

Coordinating the wide variety of sectors and actors that are implicated in tackling malnutrition is extremely challenging, and high-level multisectoral coordination mechanisms are required at the country level. There is evidence that the countries of the region recognize the importance of a multisectoral approach to tackle malnutrition. The majority (86%) report having one or more multisectoral coordination mechanisms for nutrition [27]. 

Four sectors—health, agriculture, education and water/sanitation—play a particularly key role, and national nutrition policies and strategies should address the role of these sectors.

#### 3.2.1. Health

Throughout the region, the health sector is driving action on nutrition—health ministries were involved in all the multisectoral coordination mechanisms across the region, and in the majority of cases (88%), such mechanisms are located within the health ministry [27]. 

Within the health system, all the essential nutrition actions described by WHO need to be mainstreamed into service provision [28]. Universal access to adequate antenatal and postnatal care in health facilities is essential. Strong and resilient health systems, which are accessible to all, are needed to be able to deliver nutrition interventions, protect maternal health—starting with adolescent girls—and promote optimal infant and young child feeding. Policies and programs are needed for the prevention and control of malaria and other infectious diseases, to improve treatment of infectious diseases and provide micronutrient supplements, as appropriate. Furthermore, legislative measures to regulate the marketing of breast milk substitutes, end inappropriate promotion of foods for infants and young children and require employers to provide paid maternity leave and breastfeeding breaks are important. 

#### 3.2.2. Education

Across the region, there is some recognition of the important role of education in nutrition policy—with the education ministry involved in more than half (55%) of multisectoral coordination mechanisms for nutrition—but there remains room for improvement [27]. 

There are a variety of ways in which education can help address the causes of stunting. Caregiver education is an important determinant of child health and nutritional status [38]. This is especially true of mothers and, thus, policies that expand girls’ access to education, including throughout adolescence, are important [39], with positive health outcomes for young women and their children. Incorporating nutrition and hygiene into school curricula provides an opportunity to teach future parents about healthy diets and good hygienic practice. 

Beyond the formal education sector, a variety of educational techniques, from individual counseling to mass media, can be used to promote behavior change messages relating to infant feeding practices—including breastfeeding and complementary feeding—hygiene and caregiving, including giving children opportunities for play and learning.

#### 3.2.3. Agriculture

The agriculture sector is involved in less than half (45%) of the nutrition coordination mechanisms among the countries of the region [27]. This is despite the fact that access to a diverse range of micronutrient-rich foods, particularly during the complementary feeding period, is vital for the prevention of stunting [40,41]. Globally, and in the region, there is a need to transform agriculture to build food systems that provide healthy diets [13,42]. Major efforts are needed to increase the diversity of the food supply in a sustainable way [42]. This can be achieved, for example, by supporting local food producers and small family farms and creating incentives for greater production of nutrient-rich foods. Food safety is also critical for improved nutrition and prevention of food-borne infections, so investment in safe storage and handling, effective food control systems and robust food safety standards are needed [43].

#### 3.2.4. Water and Sanitation

Frequent infection and poor nutrition interact and can exacerbate one another in a downward spiral of worsening nutritional status and greater susceptibility to infection [39]. It has also been suggested that sub-clinical infection contributes to poor growth and development [39]. A lack of access to sanitation and to safe drinking water, exposure to other environmental contaminants and poor hygiene practices can all contribute [38]. Water, sanitation and hygiene (WASH) actions or interventions were included in the nutrition policy, strategy or plans of 10 countries in the region, according to the analysis of data collected for the Global Nutrition Policy Review 2016–2017 [27]. 

## 4. Discussion

Estimates from UNICEF, WHO and the World Bank show that the prevalence of child stunting in the WHO Eastern Mediterranean Region has fallen dramatically in the last three decades—dropping from 40% in 1990 to 24.2% in 2019. The absolute numbers of children affected by stunting also declined between 1990 and 2019 (from 24.5 to 20.6 million), but much more dramatic progress will be needed to achieve the global target to reduce the number of children affected by stunting by 40% from 2012 levels by 2025 [29] or global and regional targets for a 50% reduction by 2030 [13,14]. Prevalence varies considerably between countries and there is an inverse relationship between stunting prevalence and country income and level of human development.

### 4.1. Priority Actions to Tackle Stunting

Across the different sectors described above, several areas of action can be highlighted for implementation at scale to reduce stunting. Many of the recommended actions will also contribute to achieving other nutrition goals and targets—such as to reduce prevalence of wasting, low birth weight and anemia, halting the increase in childhood overweight and increasing breastfeeding—as well as some of the global targets for the prevention and control of NCDs. As such, all of these actions belong as part of a comprehensive nutrition strategy and action plan to tackle all forms of malnutrition.

As the results above highlight, there is an ongoing need to improve the collection and reporting of data on linear child growth. Only 11 of the region’s 22 Member States have sufficient quality data to be able to make a meaningful assessment of progress towards the global and regional nutrition targets. The lack of systematic, regular conduct of national nutrition surveys results in limitations on available data to track progress and to enable comparisons, and it can be particularly challenging to obtain quality data on the most vulnerable populations. It is important to incorporate a linear growth assessment into routine child health services [4] and sometimes field work to assess the situation of the most hard-to-reach populations is needed. The WHO Eastern Mediterranean regional nutrition strategy calls on countries to establish or strengthen food and nutrition surveillance systems [13], and technical guidance is available to help countries improve monitoring and surveillance [21,44]. 

Creation of healthy food environments that enable people to access affordable diversified, balanced and healthy diets is important for preventing all forms of malnutrition [28]. The WHO regional nutrition strategy calls for urgent action to reform food systems to improve production of, and access to, foods which comprise healthy diets, including by strengthening local food supply chains and increasing the diversity of food production in a sustainable way [13]. It also includes priority actions to ensure provision of healthy food in schools, hospitals and other public institutions, implementation of best practice on wheat flour fortification and mandatory standards for food labeling, including simplified front-of-pack labeling.

Policies and interventions to improve maternal nutrition and health, beginning with adolescent girls, are important for tackling stunting [4]. Improving maternal nutrition is absolutely key to breaking the intergenerational cycle of malnutrition, including specifically reducing stunting. This requires a multisectoral and life-cycle approach, as set out in the WHO Comprehensive Implementation Plan on Maternal, Infant and Young Child Nutrition [29]. Essential nutrition actions (see Table 2) should be integrated into health systems, and universal access should be ensured. Nutrition-sensitive social protection policies can also play a role in ensuring that women and girls receive adequate food. These are both highlighted as priority actions in the regional nutrition strategy [13].

Interventions for improved exclusive breastfeeding and complementary feeding practices are needed to promote optimal infant and young child feeding, which is, in turn, absolutely key to efforts combatting stunting and other forms of malnutrition [28,29]. Early initiation of breastfeeding and exclusive breastfeeding for six months meets babies’ nutrient needs and provides vital protection against gastrointestinal infections that can lead to nutrient depletion and stunting. Continued breastfeeding up to the age of two or beyond makes a significant contribution to key nutrient needs. It is also critical to ensure that complementary feeding is timely, adequate, safe and appropriate. This can often require efforts to increase dietary diversity by improving the availability and affordability of a diverse range of nutrient-rich foods. Reinforcing the package of policies and interventions to promote, protect and support breastfeeding and appropriate complementary feeding is recommended as a priority in the regional nutrition strategy [13].

Community-based interventions, including improved water, sanitation and hygiene (WASH), need to be strengthened to protect children from diarrheal diseases and malaria, intestinal worms and environmental causes of subclinical infection [4]. Culturally appropriate policies to ensure universal access to sanitation and clean drinking water are important, as well as information and education to promote good hygiene practices, including handwashing with soap [28].

### 4.2. Country Experience in Reducing Stunting

Examination of country case studies shows that sizeable reductions in stunting prevalence can be achieved and points to the importance of multisectoral action.

In Iran, to take one example, a multisectoral intervention to address child malnutrition was implemented involving components relating to health, education, agriculture, water and sanitation in three rural districts in the late 1990s [45]. This included, among other things: educating caregivers about breastfeeding, complementary feeding and hygiene; reinforcing the growth monitoring program; strengthening literacy programs for women; establishing rural cooperatives to increase access to foods; granting loans for family self-sufficiency; and establishing employment and income generation programs. To promote breastfeeding and complementary feeding, pre- and postnatal counseling was provided by women health volunteers (Behvarz), who also demonstrated food preparation and provided village health facilities (health houses) with gas cookers for demonstrations. To improve hygiene and prevent infectious disease, the Behvarz coordinated activities to expand access to safe drinking water and toilet structures, as well as improved procedures for waste collection and disposal. To encourage increased vegetable production and consumption, seeds were distributed to village health houses and schools, while women and girls were educated on nutrition. Between 1996 and 1999, the prevalence of stunting declined considerably in the three intervention areas (from 25% to 12% in Ilan, 41% to 13% in Borazjan and 31% to 19% in Bardsir), while there were much smaller decreases in control areas. Nationally, prevalence of stunting in children under 5 declined from 24.4% in 1995 to 6.8% in 2011 [1].

Although the prevalence of stunting in Morocco is still considered to be of medium public health significance, it is another example of a country where prevalence has reduced, from 28% in 1987 to 15.1% in 2018 [46], and where a variety of policies and interventions have been implemented. Screening and management of malnutrition was introduced, and the WHO recommended standards for growth monitoring have been adopted since 2008. Other interventions included micronutrient supplementation of children and women, fortification of widely consumed foods with micronutrients [47], supply of drinking water to rural (97.4%) and urban areas (100%) in 2018 and a nutrition education program on feeding children under 5 and improvement of the quality of care for children. In addition, since 2011, a package of interventions has been implemented to promote and support breastfeeding and infant and young child nutrition. These include revitalization of the Baby Friendly Hospital Initiative, a social mobilization plan to involve other sectors, institutionalization of the National Breastfeeding Promotion Week, building health professional skills for counseling on infant and young child feeding, awareness-raising sessions for mothers in health facilities, prohibition of free distribution of infant food and, since 2012, compliance by public health structures with a ministerial circular implementing the International Code of Marketing of Breast-milk Substitutes. The prevalence of early breastfeeding increased from 26% in 2011 to 42.6% in 2018, the average duration of breastfeeding increased from 13.9 months in 2003 to 17.4 months in 2018 [47] and the prevalence of exclusive breastfeeding among infants 0–5 months increased from 27.8% to 35% between 2011 and 2018 [48]. However, caregivers’ understanding of dietary diversification in children over 6 months is still inadequate. Only one in five children aged 6 to 11 months receives an appropriate diet [47]. With the nutrition transition affecting both urban and rural areas, there is increasing consumption of highly processed foods poor in micronutrients and a shift away from micronutrient-rich foods. Other relevant interventions include improving the adult literacy rate through the strengthening of literacy programs [49] and supporting development of agricultural cooperatives to promote social and economic development in rural areas. 

### 4.3. Tackling Stunting and Other Forms of Malnutrition

Stunting is only one form of childhood malnutrition, and a fuller picture of infant and young child nutrition in the Eastern Mediterranean Region should also consider prevalence of moderate and severe wasting, low birth weight, childhood overweight and micronutrient deficiencies. The countries of the WHO Eastern Mediterranean Region—which spans from Morocco to Pakistan—are incredibly diverse in their socioeconomic status and the health challenges that they face [13]. This diversity is reflected in the nutrition situation of different countries.

Food insecurity and acute malnutrition remain a problem across the region—around 55 million people in the Region suffer from hunger (food insecurity) [50] and 6.4 million children under 5 (equivalent to 7.5% of the under 5 population) were severe or moderately wasted in 2019, 2.6 million of whom were severely wasted. A number of countries in the region are experiencing or recovering from conflict- or instability-related humanitarian emergencies (Afghanistan, Iraq, Syria, Libya, Palestine, Sudan, Somalia and Yemen). Some of the world’s most serious food crises—even prior to the emergence of COVID-19—are in the region: namely, Sudan, Syria and Yemen [51]. 

It is estimated that 17% of babies born in the region in 2015 were low birth weight, representing 2.9 million newborns [52]. The prevalence has decreased slightly from 19.4% in 2000, although the numbers of babies affected has increased from 2.7 million. 

In 2019, 5.7% of children under 5 in the region were estimated to be overweight, with serious implications for their health and well-being throughout the life course. Prevalence at the regional level has not increased since 1990 (6.2%)—and has remained stable throughout the last decade—but the absolute number of children affected has increased from 3.8 million to 4.9 million and there is considerable variation between countries. Overweight and obesity have increased in older children, adolescents and adults, reflecting the nutrition transition involving a shift towards unhealthy diets and sedentary lifestyles experienced by most countries in the region. Despite the diversity of countries in the region, it is a general characteristic that countries are experiencing the double burden of malnutrition, faced with undernutrition, at the same time as increasing rates of overweight, obesity and diet-related NCDs. 

Given this overall picture, there is a need to integrate efforts to combat stunting into comprehensive food and nutrition strategies to tackle all forms of malnutrition, improve nutrition and ensure access to healthy diets. There are many co-benefits to be had from interventions to address stunting for other areas of nutrition. Improving breastfeeding and complementary feeding, for example, is vital for tackling stunting and for the prevention of overweight and obesity. There is potential for synergies between efforts to meet the global nutrition targets—interventions targeted at increasing breastfeeding, reducing anemia in women of reproductive age and reducing low birth weight could all help to reduce stunting.

This approach is embedded in the WHO Strategy on nutrition for the Eastern Mediterranean Region 2020–2030 [13]. The priority actions for tackling stunting outlined above are well aligned with the proposed priority actions in the Strategy, which, in turn, reflect the six key areas of action of the United Nations Decade of Action on Nutrition 2016–2025.

### 4.4. Barriers and Challenges to Nutrition Action in the Context of COVID-19

There are significant barriers and challenges facing countries in their efforts to make further progress to reduce childhood stunting. As noted previously, several countries in the region have been badly affected by conflict and face emergency situations and/or long-term development challenges. Investment in nutrition requires resources and human capacity, and these are constrained in many countries. Furthermore, a lack of nutrition and health data, sometimes coupled with weak governance and poorly defined responsibility and accountability, can hinder progress. 

Countries in the region have scarcely had the opportunity to begin operationalizing the new regional strategy than the COVID-19 pandemic has thrown up new barriers and challenges [53]. In the short term, this may lead to increased prevalence of food insecurity and acute malnutrition (wasting). Preliminary estimates suggest that the pandemic may add between 83 and 132 million people to the total number of undernourished people in the world in 2020 [42]. Regional modeling estimated that between 2000 and 12,000 children under 5 could die in six months in 2020 in the Food and Agriculture Organization of the United Nations (FAO) Near East and North Africa Region due to wasting caused by the impact of the COVID-19 pandemic on food access and healthcare [54]. There are also risks, however, that the combined effects of COVID-19 and the measures taken to mitigate its impact, along with the emerging global recession, will increase other forms of malnutrition, including stunting, through their effects on access and affordability of safe and nutritious foods and access to essential health services. In situations of economic difficulty, families may shift away from relatively more expensive nutrient-rich foods to cheaper sources of calories. Healthy diets are, on average, more expensive than diets that just meet needs for energy and other nutrients and are already unaffordable for many [42]. Moreover, disruptions to food supply chains may also hinder access to diverse, healthy foods. The FAO has issued advice on policy action and interventions to maintain food supply chains and protect access to foods in the region [51]. Disruption to health services is likely to impact on the delivery and continuity of essential actions for maternal, infant and young child nutrition. Although it was adopted before the pandemic, the new WHO regional nutrition strategy provides a framework to reinforce the protection of food security and nutrition from the impact of COVID-19.

A regional consultation organized by the WHO Regional Office in January 2019 identified a number of support needs for implementation of the new regional nutrition strategy. Those that are particularly relevant to efforts to reduce stunting included support on nutrition surveillance systems, help to draft relevant regulatory instruments and establishment of platform(s) to enable and encourage exchange and learning between countries [13].

## 5. Conclusions

As the COVID-19 pandemic threatens to undermine nutrition and food security globally and in the Eastern Mediterranean Region, it is more important than ever to adapt and implement multisectoral responses to protect the children of the region from malnutrition. Real progress has been achieved in reducing the rate of stunting in recent decades, but much faster reductions will be needed for the region to meet global and regional targets. The necessary policies, interventions and tools to tackle stunting are known and available and belong as part of comprehensive strategies to address malnutrition in all its forms.

## Figures and Tables

**Table 1:** Prevalence of stunting among children under 5 in countries of the WHO Eastern Mediterranean Region (%, baseline and latest available data) ^1^, and pre-baseline and current average annual rate of reduction (AARR).

Country	Baseline (% (Year))	Latest (% (Year))	Pre-Baseline AARR	Current AARR	Required AARR to Reach the 2025 Target
Afghanistan	40.4 (2013)	38.2 (2018)		1.1	4.4
Bahrain	(a)				
Djibouti	33.5 (2012)	33.5 (2012)	−2.1		4.2
Egypt	30.7 (2008)	22.3 (2014)	−3.3	5.2	5.6
Iran	6.8 (2011)	6.8 (2011)	0.6		4.4
Iraq	22.1 (2011)	12.6 (2018)	2.3	7.7	5.3
Jordan	7.8 (2012)	7.8 (2012)	4.2	1.7	3.2
Kuwait	4.3 (2012)	6.4 (2017)	−0.8	−4.5	2.5
Lebanon	(a)				
Libya	21 (2007)	38.1 (2014)	5.7		3.7
Morocco	14.9 (2011)	15.1 (2017)	5.3	−0.2	3.8
Oman	9.8 (2009)	11.4 (2017)	4.7	−2.5	6.4
Occupied Palestinian Territory	10.9 (2010)	7.4 (2014)	3.4	9.2	4.8
Pakistan	43 (2011)	37.6 (2018)	−0.4	2.2	4.9
Qatar	(a)		5.7		
Saudi Arabia	9.3 (2005)		5.7		4
Somalia	25.3 (2009)	25.3 (2009)	0.5		6.1
Sudan	34.1 (2010)	38.2 (2014)	2.9	−2.9	5.2
Syria	27.9 (2010)	27.9 (2010)	−0.4		2.6
Tunisia	10.1 (2012)	8.4 (2018)	4.2	3.0	3.8
United Arab Emirates	No data	No data			
Yemen	46.6 (2011)	46.4 (2013)	2.7	0.2	4.7

^1^ Source: data from the UNICEF/WHO/World Bank joint malnutrition estimates dataset [30]; (a) latest data older than 2005, thus no baseline for assessing progress towards the target.

**Table 2:** Essential nutrition actions for reducing the prevalence of stunting.

Area of Action	Specific Interventions
Promoting healthy diets	Create a healthy food environment that enables people to adopt and maintain healthy dietary practices
Protecting, promoting and supporting breastfeeding	Support early initiation, establishment and maintenance of breastfeeding and immediate skin-to-skin contact Optimize newborn feeding practices and address additional care needs of infants Create an enabling environment for breastfeeding in health facilities Enable exclusive breastfeeding for the first 6 months of life Enable continued breastfeeding Counsel women to improve breastfeeding practices
Care of low-birth weight and very low birth weight infants	Optimal feeding of low-birth weight and very low birth weight infants Enable kangaroo mother care for low-birth weight infants
Appropriate complementary feeding	Enable feeding of appropriate complementary foods
Growth monitoring and assessment	Weight and height or length assessments for children under 5 years of age Nutrition counseling for children under 5 years of age Develop a management plan for overweight children under 5 years of age presenting to primary healthcare facilities
Vitamin A supplementation	High-dose vitamin A supplementation for infants and children aged 6–59 months
Zinc supplementation in the management of diarrhea	Zinc supplementation with increased fluids and continued feeding for management of diarrhea in children
Nutritional care of women during pregnancy and postpartum	Nutritional counseling on healthy diet to reduce the risk of low birth weight Energy and protein dietary supplements for pregnant women in undernourished populations Daily iron and folic acid supplementation for pregnant women Intermittent iron and folic acid supplementation for pregnant women Vitamin A supplementation for pregnant women
Specific conditions	Ensure optimal infant and young child feeding for infants of mothers infected with tuberculosis, Ebola virus disease, viral hemorrhagic disease or who are carriers of hepatitis B and in the context of HIV or Zika virus transmission and areas with an ongoing pandemic of influenza A (H1N1)
Emergencies	Ensure optimal infant and young child feeding in emergencies (and micronutrient supplementation as appropriate); nutritional support and micronutrient supplementation for pregnant and lactating women affected by an emergency

Source: adapted from Essential nutrition actions: mainstreaming nutrition through the life-course [28].
